# Supplementary material for: Chromothripsis during telomere crisis is independent of NHEJ, and consistent with a replicative origin
Source: Genome Res. 2019 May;29(5):737–49. doi: 10.1101/gr.240705.118 (PMC6499312; doi:10.1101/gr.240705.118)
Supplement: Supplemental Material [file supp_gr.240705.118_Supplemental_file_1.zip › contigs/annotated_contigs/DB92/contig.2.DB92_length_504_mean_cov_7.10714285714.docx]

**DB92_length_504_mean_cov_7.10714285714**

```
GGGCTCAAGCAATCCTCCCATCTCAGCCCCTAGAGTAGCTGGGACTACAGGTGCACGCCAACATGCCCAGCTAATTTTTTTGGATTTCT
 >chr11:111915439-111915683 - E=3e-135 p=4e-02
AGTAGAGACGGAAATTAGCCACGTTGCCCTGGCTGGTCTCAAATGCCTGGGCTCAAGTGATCCACCCACCTTGGCTTCCCAAAGTGCTG

GGGTTACAAGCATGAGCCACCATGTCCAGTCTAAAAACTAAGCTTTATAGAATACCTATTTCTG|TC|ACAGGTTGGGTGCAGTGGCTC
                                                                 >chr11:112033110-11203
ACTCCTGTAATCCCAACACTTTGGAAGGCCAAGGCAGGTGGATTGCTTCAGCTCAGGAGTTCAAGACCAGCCTGGGCAACATGGCAAAA
3372 - E=4e-143
ACCCTGTCTCTCCAAAACATACAAAAATTAGCCAGATGTGGTAGTATGTGCCTGTAGTCCCAACTACTCAAAAGGCTAAGGCAAGAGGA

TCACTTGAGCCCAGGAGGTCAAGGCTACAGTGAGCCATGTTTACTGTGTCACTGCACTCCA
```
